# South Asia as a Reservoir for the Global Spread of Ciprofloxacin-Resistant *Shigella sonnei*: A Cross-Sectional Study

**DOI:** 10.1371/journal.pmed.1002055

**Published:** 2016-08-02

**Authors:** Hao Chung The, Maia A. Rabaa, Duy Pham Thanh, Niall De Lappe, Martin Cormican, Mary Valcanis, Benjamin P. Howden, Sonam Wangchuk, Ladaporn Bodhidatta, Carl J. Mason, To Nguyen Thi Nguyen, Duong Vu Thuy, Corinne N. Thompson, Nguyen Phu Huong Lan, Phat Voong Vinh, Tuyen Ha Thanh, Paul Turner, Poda Sar, Guy Thwaites, Nicholas R. Thomson, Kathryn E. Holt, Stephen Baker

**Affiliations:** 1 The Hospital for Tropical Diseases, Wellcome Trust Major Overseas Programme, Oxford University Clinical Research Unit, Ho Chi Minh City, Vietnam; 2 Centre for Tropical Medicine and Global Health, Oxford University, Oxford, United Kingdom; 3 National *Salmonella*, *Shigella*, and *Listeria monocytogenes* Reference Laboratory, University Hospital Galway, Galway, Ireland; 4 School of Medicine, National University of Ireland Galway, Galway, Ireland; 5 Microbiological Diagnostic Unit Public Health Laboratory, Department of Microbiology and Immunology, Peter Doherty Institute for Infection and Immunity, The University of Melbourne, Melbourne, Australia; 6 Public Health Laboratory, Department of Public Health, Ministry of Health, Royal Government of Bhutan, Thimphu, Bhutan; 7 Department of Enteric Diseases, Armed Forces Research Institute of Medical Sciences, Bangkok, Thailand; 8 The Hospital for Tropical Diseases, Ho Chi Minh City, Vietnam; 9 Cambodia-Oxford Medical Research Unit, Angkor Hospital for Children, Siem Reap, Cambodia; 10 The London School of Hygiene and Tropical Medicine, London, United Kingdom; 11 The Wellcome Trust Sanger Institute, Hinxton, Cambridgeshire, United Kingdom; 12 Centre for Systems Genomics, The University of Melbourne, Melbourne, Australia; 13 Department of Biochemistry and Molecular Biology, Bio21 Molecular Science and Biotechnology Institute, University of Melbourne, Melbourne, Australia; Mahidol-Oxford Tropical Medicine Research Unit, THAILAND

## Abstract

**Background:**

Antimicrobial resistance is a major issue in the *Shigellae*, particularly as a specific multidrug-resistant (MDR) lineage of *Shigella sonnei* (lineage III) is becoming globally dominant. Ciprofloxacin is a recommended treatment for *Shigella* infections. However, ciprofloxacin-resistant *S*. *sonnei* are being increasingly isolated in Asia and sporadically reported on other continents. We hypothesized that Asia is a primary hub for the recent international spread of ciprofloxacin-resistant *S*. *sonnei*.

**Methods and Findings:**

We performed whole-genome sequencing on a collection of 60 contemporaneous ciprofloxacin-resistant *S*. *sonnei* isolated in four countries within Asia (Vietnam, *n* = 11; Bhutan, *n* = 12; Thailand, *n* = 1; Cambodia, *n* = 1) and two outside of Asia (Australia, *n* = 19; Ireland, *n* = 16). We reconstructed the recent evolutionary history of these organisms and combined these data with their geographical location of isolation. Placing these sequences into a global phylogeny, we found that all ciprofloxacin-resistant *S*. *sonnei* formed a single clade within a Central Asian expansion of lineage III. Furthermore, our data show that resistance to ciprofloxacin within *S*. *sonnei* may be globally attributed to a single clonal emergence event, encompassing sequential *gyrA*-S83L, *parC*-S80I, and *gyrA*-D87G mutations. Geographical data predict that South Asia is the likely primary source of these organisms, which are being regularly exported across Asia and intercontinentally into Australia, the United States and Europe. Our analysis was limited by the number of *S*. *sonnei* sequences available from diverse geographical areas and time periods, and we cannot discount the potential existence of other unsampled reservoir populations of antimicrobial-resistant *S*. *sonnei*.

**Conclusions:**

This study suggests that a single clone, which is widespread in South Asia, is likely driving the current intercontinental surge of ciprofloxacin-resistant *S*. *sonnei* and is capable of establishing endemic transmission in new locations. Despite being limited in geographical scope, our work has major implications for understanding the international transfer of antimicrobial-resistant pathogens, with *S*. *sonnei* acting as a tractable model for studying how antimicrobial-resistant Gram-negative bacteria spread globally.

## Abstract 2

Stephen Baker and colleagues use whole-genome sequencing and phylogenetic charactarization of a global collection of ciprofloxacin-resistant *Shigella sonnei* isolates to explore the origins of the recent spread of the antimicrobial-resistant infection.

## Author Summary

**Why Was This Study Done?:**

- Antimicrobial resistance is a major issue in *Shigella*, and ciprofloxacin is a recommended treatment for *Shigella* infections.
- Ciprofloxacin-resistant *Shigella sonnei* are being increasingly isolated globally.

**What Did the Researchers Do and Find?:**

- We performed genome sequencing on 60 ciprofloxacin-resistant *S*. *sonnei* isolated in six countries within and outside of Asia.
- Placing these genome sequences in context with other strains, we found that all ciprofloxacin-resistant *S*. *sonnei* formed a single clade, with South Asia as the likely primary source of these organisms.
- These organisms are also being regularly exported across Asia and intercontinentally into Australia, the United States, and Europe.
- The number of *S*. *sonnei* sequences available from other locations limited our analysis.

**What Do These Findings Mean?:**

- Our work suggests that a single clone, which is widespread in South Asia, is driving the current global surge of ciprofloxacin-resistant *S*. *sonnei*.
- We argue that *S*. *sonnei* acts as a tractable model for studying how antimicrobial-resistant, Gram-negative bacteria spread globally.

## Introduction

Diarrheal disease is the second most common cause of mortality in children under the age of 5 y worldwide, equating to approximately 800,000 deaths per year [1]. The recent Global Enteric Multicentre Study (GEMS), a large, prospective, case-control study focusing on mild and severe paediatric diarrheal illnesses in sub-Saharan Africa and South Asia, found that *Shigella* (a genus of Gram-negative enteric bacteria) were amongst the top four most prevalent diarrhoeal pathogens in these settings [2]. The most recent estimates suggest that *Shigella* infections account for around 125 million cases of diarrhoea annually, with the majority occurring in children in low-income countries [3]. There are four *Shigella* species (*dysenteriae*, *boydii*, *flexneri*, and *sonnei*), but the overwhelming majority of the current global burden is presently caused by *S*. *sonnei* and *S*. *flexneri*. Present-day international epidemiology of the various *Shigella* species is particularly intriguing, as *S*. *sonnei* is replacing *S*. *flexneri* as the most common cause of shigellosis worldwide; this pattern is accentuated in regions undergoing rapid economic development [4,5], where *S*. *flexneri* dominated as recently as a decade ago.

*Shigella* infections are characterised by the invasion and disruption of the epithelial cells lining the gastrointestinal mucosa, resulting in mucous and/or bloody diarrhoeal discharge. Although shigellosis is typically self-limiting, antimicrobial treatment is used to prevent complications, reduce dysenteric discharge, and curb post-symptomatic faecal shedding [6,7]. Consequently, resistance to antimicrobials restricts treatment options, placing vulnerable individuals suffering from shigellosis at increased risk of complications and increasing the likelihood of protracted faecal shedding. One of the current recommended first-line treatments for shigellosis is the fluoroquinolone ciprofloxacin [8]. The fluoroquinolones target the DNA gyrase, a type II topoisomerase that is essential for bacterial DNA replication and transcription [9].

Antimicrobial resistance is an emerging global issue in *S*. *sonnei*, with a specific multidrug-resistant (MDR) lineage (III) now dominating internationally. Furthermore, organisms belonging to lineage III appear to be highly proficient at acquiring resistance to additional antimicrobials (including third-generation cephalosporins) when they are introduced into new locations [10]. However, given their common usage and broad spectrum of activity, resistance against the fluoroquinolones is the most concerning. Since the first isolation of *S*. *sonnei* with reduced susceptibility to ciprofloxacin in Japan in 1993 [11], ciprofloxacin-resistant *S*. *sonnei* have been increasingly reported throughout Asia [12–14]. Furthermore, public health laboratories in several non-Asian countries with low incidences of shigellosis have reported the isolation of ciprofloxacin-resistant *S*. *sonnei*, often from individuals reporting recent travel to locations with a high risk of shigellosis [15–17].

Whole-genome sequencing has proven to be the gold standard for tracking the international dissemination of clonal bacterial pathogens [18,19], and we have previously exploited this method to study the phylogenetic structure and spread of *S*. *sonnei* at both national and intercontinental levels [10,20]. Hypothesising that Asia was a hub for the recent international spread of ciprofloxacin-resistant *S*. *sonnei*, we performed whole-genome sequencing and phylogenetic characterisation of a collection of ciprofloxacin-resistant *S*. *sonnei* isolated from within and outside Asia, aiming to explore the origins of this growing international epidemic.

## Methods

### Ethics Statement

*S*. *sonnei* isolates from Bhutan, Thailand, and Vietnam were collected during diarrheal surveillance studies [14]. IRB approval was granted for these studies (including organism characterization) from the Research Ethics Board of Health (REBH), Ministry of Health, Bhutan (Bhutan study), the Walter Reed Army Institute of Research (WRAIR) Institutional Review Board, USA (Bhutan and Thailand studies), the Oxford Tropical Research Ethics Committee (OxTREC), UK, and the Hospital for Tropical Diseases Ho Chi Minh City, Vietnam (Vietnam study). Written (Vietnam) or oral (Thailand and Bhutan) consent was obtained from a parent or guardian at the time of enrolment into the study. The target patient groups for these studies were generally hospitalised children aged less than 5 y residing in close proximity to the study centres. *S*. *sonnei* isolates from Cambodia were collected at the Angkor Hospital for Children (AHC) in Siem Reap province from the routine diagnostic laboratory; no patient data were collected, and these organisms were not subject to local or international IRB approval. The ciprofloxacin-resistant *S*. *sonnei* from countries outside Asia were collected and characterized by the National *Salmonella*, *Shigella*, and *Listeria monocytogenes* Reference Laboratory, Galway, Ireland, and the Microbiological Diagnostic Unit Public Health Laboratory, Melbourne, Australia. These isolates were generally, but not exclusively, obtained from patients reporting recent travel to countries with a high incidence of shigellosis in Asia (Table 1). As these isolates were from anonymous sources and collected at local public health laboratories, these were not subject to IRB approval, and informed consent was not required.

**Table 1:** The origins of the *Shigella sonnei* isolates and sequences used in this study.

Country	Isolates in Central Asia clade (N)	Ciprofloxacin-resistant isolates (N)	Study or institute origin	IRB approval or public database access	Patient group	Region of recent travel history (N)	Sequencing platform/Public database	Ciprofloxacin susceptibility
Bhutan	12	12	Diarrhoeal disease surveillance in JDWNRH^a^, Thimphu, Bhutan (AFRIMS^b^)	The Research Ethics Board of Health (REBH), Ministry of Health, Bhutan, and the Walter Reed Army Institute of Research (WRAIR) Institutional Review Board, USA	Hospitalised children <5 y old	NA	Illumina HiSeq 2000	Disk diffusion/E-test
Vietnam	11	11	Diarrhoeal disease surveillance in Ho Chi Minh City, Vietnam (OUCRU^c^)	Oxford Tropical Research Ethics Committee (OxTREC), UK, and the Hospital for Tropical Diseases Ho Chi Minh City, Vietnam	Hospitalised children <5 y old	NA	Illumina MiSeq	Disk diffusion
Thailand	8	1	Diarrhoeal disease surveillance in Thailand (AFRIMS)	The Research Ethics Board of Health (REBH), Ministry of Health, Bhutan, and the Walter Reed Army Institute of Research (WRAIR) Institutional Review Board, USA	Hospitalised children <5 y old	NA	Illumina HiSeq 2000	Disk diffusion
Cambodia	1	1	Diarrhoeal disease surveillance in Siem Reap, Cambodia (COMRU^d^)	Anonymous clinical isolates collected as part of routine diagnosis—IRB approval not required	Hospitalised children <5 y old	NA	Illumina HiSeq 2000	Disk diffusion
Ireland	20	16	National *Salmonella*, *Shigella*, and *L*. *monocytogenes* Reference Laboratory, Galway, Ireland	Anonymous clinical isolates sent to public health reference laboratory—IRB approval not required	Primarily patients with recent travel history	India (9), Germany (1), Morocco (1), No travel (5), Unknown (4)	Illumina HiSeq 2000	Broth microdilution
Australia	20	19	Microbiological Diagnostic Unit Public Health Laboratory in Melbourne, Australia	Anonymous clinical isolates sent to public health reference laboratory—IRB approval not required	Patients with recent travel history	India (15), Cambodia (3), Thailand (1), Southeast Asia (1).	Illumina NextSeq	Agar dilution
United States	14	10	Genome Trackr; Centre for Disease Control and Prevention, USA	Data accessed from NCBI under the BioProject accession number PRJNA218110	Unknown	Unknown	Illumina MiSeq	in silico assessment of QRDR^e^ mutations

^a^ Jigme Dorji Wangchuk National Referral Hospital

^b^ Armed Forces Research Institute of Medical Sciences

^c^ Oxford University Clinical Research Unit

^d^ Cambodia Oxford Medical Research Unit

^e^ quinolone resistance-determining region

NA, Not applicable

### Strain Collection

Aiming to investigate the current international upsurge in ciprofloxacin-resistant *S*. *sonnei* in detail, we gathered a collection of 60 contemporary ciprofloxacin-resistant *S*. *sonnei* from six countries for whole-genome sequencing. The isolates originated from Asian countries with a high incidence of shigellosis (Vietnam, *n* = 11; Bhutan, *n* = 12; Thailand, *n* = 1; Cambodia, *n* = 1), as well as countries with a low incidence of shigellosis (Australia, *n* = 19; Ireland, *n* = 16). Twelve additional ciprofloxacin-susceptible *S*. *sonnei* sequences from these settings were also included for phylogenetic context. All strains were isolated independently between 2010 and 2015; details of the isolates used in this study are shown in Table 1.

Susceptibility to ciprofloxacin was determined by disk diffusion, E-test, agar dilution, or broth microdilution, depending on the collaborating institution, and susceptibility breakpoints were interpreted according to the European Committee on Antimicrobial Susceptibility Testing (http://www.eucast.org/clinical_breakpoints). Namely, resistance was determined as strains with a zone of inhibition <19 mm (5 μg disc) and/or a minimum inhibitory concentration (MIC) >1 μg/ml against ciprofloxacin; the various location-specific methods and resulting data are described in Table 1.

### Genome Sequencing and Analysis

All isolated *S*. *sonnei* were subcultured and subjected to DNA extraction prior to whole-genome sequencing on various Illumina platforms to produce pair-ended short read sequences; the specific sequencing system and the resulting public database numbers are shown in Table 1. We additionally included 14 *S*. *sonnei* sequences from the NCBI database under the Bioproject accession number PRJNA218110. These organisms were isolated in the US and deposited as part of the GenomeTrackr Project. All sequences were mapped to the *S*. *sonnei* Ss046 reference sequence (Accession number: NC_007384) using SMALT (version 0.7.4), and SNPs were called against the reference and filtered using SAMtools [21]. To contextualize all ciprofloxacin-resistant *S*. *sonnei* within the global phylogeny, we appended our collection to include 133 publicly available sequences from a previous global analysis (accession ERP000182) [20]. Previously characterized mobile genetic elements and putative recombination (predicted using Gubbins) were removed [20], resulting in a gap-free alignment of 211 non-duplicate pseudo-whole genome sequences of 4,738 SNPs. A whole-genome phylogeny was inferred from this alignment using RAxML v8.1.3 under the GTRGAMMA substitution model, and sufficient bootstrap replicates were determined automatically using the extended majority rule (MRE) bootstrap convergence criterion. Bootstrap values >75% signify strong statistical support for a node within a maximum likelihood phylogeny, thus indicating that all isolates falling within that lineage are highly likely to be linked in evolutionary history at a more recent time than those falling outside of that lineage. In order to obtain a refined phylogenetic structure of the Central Asia clade, we applied the aforementioned approach to a set of 97 *S*. *sonnei* sequences (86 novel sequences and 11 historical sequences) belonging to this clade. This resulted in an alignment of 1,121 SNPs, which was used for phylogenetic inference. De novo assemblies were generated for each read set using Velvet and VelvetOptimiser, and read sets were mapped back to each assembly [22]. A manually curated database based on ResFinder [23] was mapped against each assembly to identify mobile resistance genetic determinants in all ciprofloxacin-resistant strains.

## Results

### Fluoroquinolone-Resistant *S*. *sonnei* in a Global Context

**Fig 1:**
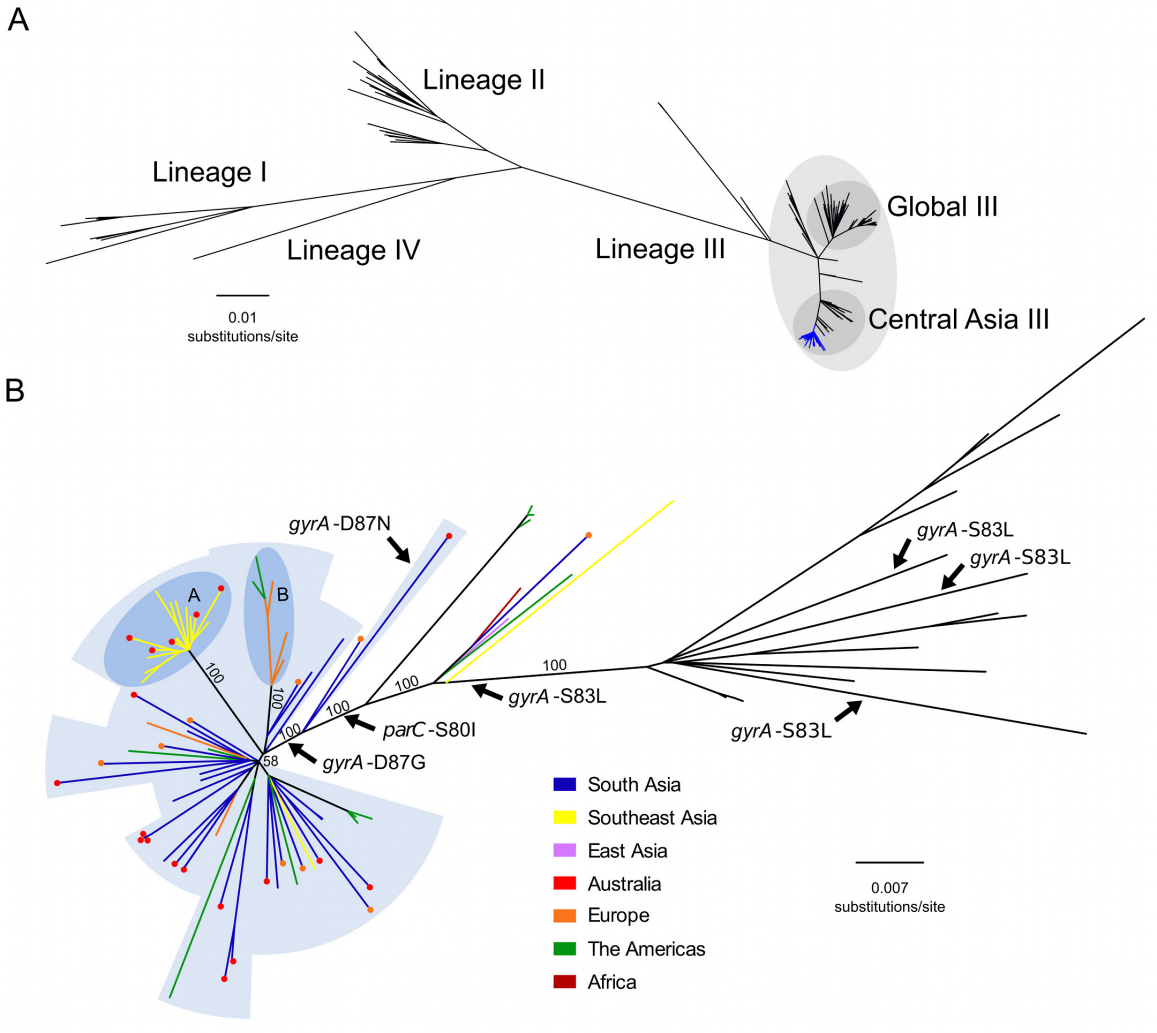
The phylogenetic structure of ciprofloxacin-resistant *Shigella sonnei* in an international context. A) Unrooted maximum likelihood phylogeny of 211 globally representative *S*. *sonnei*, including 60 sequences from ciprofloxacin-resistant isolates (highlighted by the blue branches). Major lineages are indicated by numbers (I, II, III, and IV) as defined in Holt et al. 2012, with clades Global III and Central Asia III within lineage III highlighted. Horizontal scale bar indicates the number of nucleotide substitutions per site. B) Unrooted maximum likelihood phylogeny of Central Asia III, composed of 97 *S*. *sonnei* sequences. Branch colours indicate region of isolation (where no travel history is confirmed) or region of recent travel (where travel history was confirmed) according to the keys. For isolates with confirmed recent travel, a coloured circle at the tip indicates the region where the isolate was collected (multiple coloured circles are indicative of multiple isolates). Labelled arrows indicate branches where the mutations *gyrA*-S83L, *gyrA*-D87N, *gyrA*-D87G, and *parC*-S80I have arisen. Blue background shading denotes isolates exhibiting ciprofloxacin resistance conferred by triple mutations (*gyrA*-S83L, *parC*-S80I, and *gyrA*-D87G [or *gyrA*-D87N]). Subpopulations A and B are highlighted in the darker blue shaded areas, denoting clonal expansions in Southeast Asia and Europe/America, respectively. Numbers above major branches indicate bootstrap support values, and horizontal scale bar denotes the number of nucleotide substitutions per site.

We constructed a whole genome phylogeny of *S*. *sonnei*, incorporating sequences from 133 globally representative isolates and 86 novel isolates from Vietnam, Cambodia, Thailand, Bhutan, Australia, Ireland, and the US. The novel sequences included 60 from ciprofloxacin-resistant (MIC >1 μg/ml or zone of inhibition <19 mm) organisms and 26 from ciprofloxacin-susceptible organisms (or of unknown ciprofloxacin susceptibility isolated in the US). The related metadata for the bacterial isolates are shown in S1 Table. The overall tree topology reflected the previously described global phylogenetic structure [20], confirming the presence of four distinct lineages (I, II, III, and IV); lineage III was the most commonly represented and the most widely geographically distributed (Fig 1A). All ciprofloxacin-resistant *S*. *sonnei* formed a single, well-supported monophyletic clade within the Central Asian expansion of lineage III (Central Asia III), an MDR group that is closely related but distinct from the Global III clade (Fig 1A and 1B).

### The Emergence of a Fluoroquinolone-Resistant *S*. *sonnei* Clone

We next performed a more detailed phylogenetic reconstruction of the Central Asia III clade, incorporating sequence data from the 60 phenotypically ciprofloxacin-resistant isolates and 26 others (ciprofloxacin-susceptible or of unknown ciprofloxacin susceptibility), along with 11 historical Central Asia III sequences sourced from our previous global study (Fig 1B) [20]. The majority of the Central Asia III isolates carried more than three antimicrobial resistance genes, encoding resistance to a wide range of first-line antimicrobials including tetracycline (*tetA*), streptomycin (*strAB*), and co-trimoxazole (*dfrA1 and sul2*), as previously described [20]. No plasmid-mediated quinolone resistance genes (*qnr*, *qepA*, *oxqAB*, *aac(6’)lb-cr*) were detected in the sequences of any of the ciprofloxacin-resistant *S*. *sonnei*. We additionally examined the genome sequences for mutations in the quinolone resistance-determining region (QRDR) within the DNA gyrase gene (*gyrA*) and the topoisomerase IV gene (*parC*), the regions encoding the target residues for fluoroquinolones. Overlaying these mutations on the phylogenetic tree indicated that the *gyrA*-S83L mutation, the first sequential mutation that confers reduced susceptibility against fluoroquinolones, has arisen independently within the Central Asia III clade on at least four separate occasions (Fig 1B). Amongst the isolates examined here for the first time, extensive resistance to ciprofloxacin can be attributed to a single clonal emergence event, via the sequential accumulation of *gyrA*-S83L followed by *parC*-S80I and *gyrA*-D87G, except for a single outlier isolated in Australia (Fig 1B). Strong bootstrap support at each of these nodes suggests that these mutations were sequential lineage-defining events, with the final *gyrA*-D87G mutation preceding the expansion and intercontinental dissemination of the Central Asia III clade. These three QRDR mutations were also shared by ten phenotypically uncharacterized *S*. *sonnei* from the US, thus providing genotypic evidence for ciprofloxacin resistance. The single outlier isolate shares the *gyrA*-S83L and *parC*-S80I QRDR mutations of the other ciprofloxacin-resistant isolates, but harbours *gyrA*-D87N rather than a *gyrA*-D87G, and is within a closely related out-group of the major ciprofloxacin-resistant clone (Fig 1B).

### South Asia as a Hub of Fluoroquinolone-Resistant *S*. *sonnei*

We additionally mapped the country of isolation and patient travel history onto the Central Asia III phylogeny to investigate the geographical structure of the clade (Fig 1B). For the ciprofloxacin-resistant *S*. *sonnei* isolated from countries with a low incidence of shigellosis (Ireland, Australia, and US), and for which data on recent travel history was confirmed (27/45; 60%), India was the most commonly reported travel destination (21/27; 78%). The majority of the isolates associated with travel to India clustered closely with strains isolated in neighbouring Bhutan. These data suggest that South Asia is a primary source of ciprofloxacin-resistant *S*. *sonnei* that have increasingly been isolated both within and outside of Asia in recent years. Furthermore, greater genetic diversity was observed within the South Asian *S*. *sonnei* than within the other sampled countries (Fig 1B), suggesting that this region currently acts as the most likely geographical source population.

Our data also show evidence of regional diversification of ciprofloxacin-resistant *S*. *sonnei* within Asia. The phylogenetic structure is highly suggestive of a clonal expansion of ciprofloxacin-resistant *S*. *sonnei* in Southeast Asia, specifically within Vietnam, as indicated by a long branch with 100% bootstrap support (Fig 1B). We additionally noted that *S*. *sonnei* nested within this clonal expansion were also isolated from travellers returning from countries including Cambodia and Thailand, indicating that isolates from this lineage have spread widely across Southeast Asia, as well as having been introduced into Australia on at least five separate occasions. An additional well-supported subpopulation of ciprofloxacin-resistant *S*. *sonnei*, isolated in Ireland (five individuals with no recent history of travel and one individual returning from Germany) and the US, likely represents an extended chain of local transmission within Europe and the US (Fig 1B). Alternatively, although less likely, this subpopulation could represent multiple importations and short-term local transmission of *S*. *sonnei* strains originating from an unsampled source population. Whilst it was not possible to identify the geographical source or extent of local transmission definitively, the isolates most closely related to this European/US subpopulation originated in India and Bhutan, again suggesting South Asia was the most likely origin of this subpopulation. These two expansion events in Southeast Asia and Europe/US indicate that this clone of ciprofloxacin-resistant *S*. *sonnei* is also capable of sustained circulation upon introduction into new locations.

## Discussion

Here we provide direct evidence for the ongoing global expansion of *S*. *sonnei* exhibiting new and clinically relevant antimicrobial resistance profiles. What is more, as we can use phylogeography in high resolution, we can link the reservoir of these organisms to a specific region. Therefore, this study has significant implications for understanding the international trafficking of antimicrobial-resistant bacterial pathogens from Asia. Furthermore, we suggest that, as a single-serotype, human-adapted pathogen with a clonal population structure, *S*. *sonnei* serves as a tractable model for understanding how Gram-negative antimicrobial resistant pathogens are being regularly mobilised around the globe.

To our knowledge, this is the first study that has used whole-genome sequencing to examine the emergence and global spread of ciprofloxacin-resistant *S*. *sonnei*. Our data show that all sequenced extant ciprofloxacin-resistant *S*. *sonnei*, though sourced from disparate geographical locations, belonged to a single clonal expansion of lineage III, with South Asia being the most likely hub for its origin and spread. Our findings support previous hypotheses suggesting that ciprofloxacin-resistant *S*. *sonnei* in industrialised countries is being imported from South Asia [15,16]. A recent estimation of worldwide antimicrobial usage reported that India was the largest consumer of antimicrobials in 2010 [24]. Additionally, the fluoroquinolones are ranked as the most common antimicrobial prescribed for acute enteric diseases in India and Bangladesh [25,26]. The intensive use of fluoroquinolones in a region where there are foci of high population density and inconsistent access to good sanitation is likely to have contributed to emergence of ciprofloxacin-resistant enteric bacteria, such as *S*. *sonnei* and *Salmonella* Typhi, on the Indian subcontinent [19]. Global dissemination of these organisms is likely facilitated by the volume of travel between these regions and other areas of the world.

Our new data highlight the limitations of current typing protocols for tracking *S*. *sonnei*. It had been previously observed that some of the ciprofloxacin-resistant *S*. *sonnei* isolates in this study (originating from Bhutan and Ireland) shared a similar *Xba*I pulsed field gel electrophoresis (PFGE) pattern [14,15]. This pulsotype has been observed previously in India and Bangladesh [12,13,27–29], as well as in Canada [30], Belgium [31], and Japan [32], where the association with ciprofloxacin resistance was inconsistent. However, PFGE in this context did not offer sufficient granularity to link all of the isolates or provide sufficient resolution into the regional evolution of *S*. *sonnei*. Our phylogenetic analyses show that this pulsotype is associated with a phylogenetic lineage, supporting the notion that this pulsotype actually represents a widespread and pervasive subclade of Central Asia III.

This work has limitations. First, the lack of historical organisms from South Asia restricts our inference to only the current situation. Furthermore, additional contemporary organisms from other settings would have improved our understanding of the current geographical spread of this clonal group. Although the data included in this analysis was generated from organisms sampled in diverse geographic locations, the majority of sequences were retrieved from South Asia, Southeast Asia, Europe, Australia, and the Americas. It is possible, albeit unlikely, that our study might have overlooked an undersampled population that may ultimately act as an additional reservoir. This issue is inherent to all phylogeographical investigations and can potentially be overcome by global-scale prospective sampling to capture and characterize maximal global diversity. Notwithstanding these limitations, whole-genome sequencing of these geographically disparate organisms, together with plausible epidemiological links, has provided data at the highest resolution for deciphering the emergence and international spread of ciprofloxacin-resistant *S*. *sonnei*. Future studies interrogating extensive spatial and temporal collections of ciprofloxacin-resistant *S*. *sonnei*, as well as the *S*. *sonnei* diversity specific to South Asia prior to and during the emergence of antimicrobial resistance, are essential to further elucidate the origins and epidemiological dynamics of these populations. These supplementary investigations will greatly aid our efforts in controlling the spread of the current ciprofloxacin-resistant clone and to prevent future emergent antimicrobial-resistant bacterial populations.

In conclusion, the international surge of ciprofloxacin-resistant *S*. *sonnei* clone poses a substantial global health challenge, and our data show this threat is not only manifested in sporadic cases from returning travellers but also the establishment of endemic transmission in new settings. The latter is already evident in high shigellosis incidence areas such as Southeast Asia. Therefore, integrative efforts from both the research community and public health authorities should be prioritised to track, monitor, and prevent the international spread of this key enteric pathogen.

## Supporting Information

S1 Table List of organisms used in this study for phylogenetic inference. (XLSX)

## Abbreviations

AHC: Angkor Hospital for Children
GEMS: Global Enteric Multicentre Study
MDR: multidrug-resistant
MIC: minimum inhibitory concentration
MRE: extended majority rule
OxTREC: Oxford Tropical Research Ethics Committee
PFGE: pulsed field gel electrophoresis
QRDR: quinolone resistance-determining region
REBH: Research Ethics Board of Health
WRAIR: Water Reed Army Institute of Research
